# Patients' satisfaction and opinions of their experiences during admission in a tertiary care hospital in Pakistan – a cross sectional study

**DOI:** 10.1186/1472-6963-7-161

**Published:** 2007-10-03

**Authors:** Sardar Zakariya Imam, Khezar Shahzada Syed, Syed Ahad Ali, Syed Umer Ali, Kiran Fatima, Marium Gill, Muhammad Ovais Hassan, Saad Hasan Hashmi, Maham T Siddiqi, Hadi Muhammad Khan, Omar Farooq Jameel

**Affiliations:** 1 Department of Community Health Sciences, Aga Khan University, Karachi, Pakistan

## Abstract

**Background:**

It is often felt that developing countries need to improve their quality of healthcare provision. This study hopes to generate data that can help managers and doctors to improve the standard of care they provide in line with the wishes of the patients.

**Methods:**

It was a cross sectional study carried out at a major tertiary care hospital of Karachi. Patients between the ages of 18 and 80 years admitted to the hospital for at least one day were included. Patients in the maternity, psychiatry and chemotherapy wards and those in the ICU/CCU were excluded. A pretested, peer reviewed translation of a validated patient satisfaction scale developed by the Picker Institute of Europe was administered.

**Results:**

A total of 173 patients (response rate: 78.6 %) filled the questionnaire. Patient satisfaction was at levels comparable to European surveys for most aspects of hospital care. However, nearly half the patients (48%) felt they had to wait too long to get a bed in the hospital after presenting to the ER. 68.6% of the patients said that they were never asked for views on the quality of care provided. 20% of the patients did not find anyone in the staff to talk to about their worries and fears while 27.6% felt that they were given emotional support to only some extent. Up to one third of the patients said they were not provided enough information regarding their operative procedures beforehand.

**Conclusion:**

Although several components of patient care equal the quality levels of the west, many sections require considerable improvement in order to improve health care provision. The healthcare team needs to get more involved with the patients, providing them greater support and keeping them informed and involved with their medical treatment. Efforts should be made to get regular feedback from the patients.

## Background

Provision of services in line with the wishes and needs of patients is central to a humane health care system. Society has long acknowledged the importance of the views of public in developing the very services provided to them [1] and in the case of the health care system, patients have been found to be aware of health issues to the extent that they have been described as "expert witnesses" to the health care process [2,3]. Hence over the past decade there has been increasing realization of the need to take into account patient reports of their hospital experiences in the development of action plans for improvement of services, safety and care provided. It is suggested that efforts to improve health care will be wasted unless they reflect what patients want from the service [4].

A variety of methods have therefore been employed to assess the patients' preferences for care, evaluations of what occurred, or factual reports of care. Examples are questionnaires to assess patients' needs before a consultation with the clinician, shared decision making, focus groups with patients to include their views in clinical guidelines, and surveys among patients to provide feedback to care providers or the public [5,6].

Development of newer tools and techniques to assess patient opinion is an emerging trend around the globe with the UK Patient's Charter and the review of the NHS highlighting the need for providers of hospital care to assess and improve the quality of care they offer, and to continue expanding their use of questionnaires and surveys [7,8].

This trend, however, has still not picked up in developing countries like Pakistan, where most of the 'patient satisfaction studies' still focus on specific areas such as the emergency department [9], day care surgery [10] or family medicine sections of the hospital [11]. A study is thus required to survey patients' opinions of general aspects of inpatient care provided to them during admission. Such a study becomes even more important in light of the limited budget allocation to the health sector in Pakistan and the inability of many patients to afford expensive treatment modalities. Hence there is further need to prioritize spending and this study hopes to fill this void by production of data that can help managers and doctors to identify and address unsatisfactory factors in the care they provide.

## Methods

It is a descriptive cross sectional study carried out at one of the major tertiary care hospitals in the private sector of Karachi. The hospital is also a center for undergraduate and postgraduate teaching and has an operational strength of 496 beds.

The study was performed through the months of April and May 2006. Beginning in the first week of April, 220 consecutive patients matching the inclusion criteria were included in the study and were administered the questionnaire while still being admitted in the hospital.

Patients between the ages of 18 and 80 years admitted to the Hospital since a minimum of one day were included in the study. However, patients admitted to the ICU or CCU, those admitted with conditions related to psychiatry or maternity and those undergoing chemotherapy were excluded from the study since these were considered to be exceptional circumstances. Also excluded were patients who, because of their illness were unable to communicate.

Since most of the doctors responsible for care at our hospital have obtained their qualifications from UK or Pakistan and the system of training in Pakistan is also much closer to British medical training we used a validated questionnaire [see additional file 1] designed by the Picker Institute of Europe for the NHS (National Health Services) of the United Kingdom [12]. The questionnaire was translated into Urdu by consensus of three different individuals. Two people, unfamiliar with the English version of the questionnaire back-translated the questionnaire from Urdu to English.

Expert review was carried out by two individuals belonging to the departments of Quality Assurance and Marketing. The questionnaire was then subjected to a pilot study on a convenience group of 50 patients and improved accordingly. Based on the relevance of questions to the healthcare services in Pakistan, and the results of the pilot study, our final questionnaire included 35 questions on various aspects of inpatient care. Questions were asked regarding care in the emergency section, the physical environment of the wards, doctor-patient relationship, nurse-patient relationship, quality of overall care and general treatment, as well as care related to operations and procedures. The questionnaire included points from the 15-item Picker Patient Experience questionnaire [13]. Seven additional questions pertained to the socio-demographic details of the patient.

The questionnaire was administered by trained individuals after obtaining verbal consent from all subjects. In order to maintain complete confidentiality no names were recorded on the questionnaire. Prior approval of the hospital administration was obtained before beginning the survey. The study was conducted in compliance with the *'Ethical Principles for Medical Research involving Human Subjects' *of Helsinki Declaration [14]. Verbal informed consent was obtained from all subjects.

All data was entered into and analyzed with SPSS version 13 to calculate relative frequencies and means. These were then subjectively compared to the results of surveys in other countries especially a British NHS survey [15] that utilized the same questionnaire.

## Results

A total of 220 patients meeting the inclusion criteria were included in the study. 173 patients (response rate: 78.6 %) responded to the questionnaire while 47 (21%) patients refused consent. The mean age of the sample was 46.19 years (SD: 15.94). 104 (60.1%) respondents were male while 69 (39.9%) were females.

The mean hospital stay was 5.27 days, the minimum stay being 01 day and the maximum being 60 days. Other demographic characteristics of the sample are shown in Table 1.

**Table 1:** Demographic characteristics of the sample

**Patient variable**	**Frequency (percentage)**
Monthly income (PKR)*	
< 5 000	26 (15.1%)
5–10 000	18 (10.5 %)
> 10 000	128 (74.4 %)

**Education**	
Illiterate	9 (5.2 %)
Below matric	24 (13.9 %)
Matric – graduation	91 (52.6 %)
Post-graduation	49 (28.3 %)

**Background**	
Rural	19 (11.0 %)
Urban	154 (89.0 %)

* PKR, Pakistan rupees

Table 2 shows the frequency of problems reported by the patients regarding various fields of patient care.

**Table 2:** Patients' satisfaction with the care provided to them at our hospital

**Frequency of Problems Reported by Patients**		
**Description of problem event**	**Percent of patients reporting problem**	

Emergency Section:	*Each time*	*Sometimes*
Not given enough information about condition and treatment	8.1	
Not given enough privacy during treatment and examination	5.8	40.7
Had to wait a long time before getting a bed in the ward	47.9	35.1

Ward:		
Bothered by noise at night from other patients	22.8	
Bothered by noise at night from hospital staff	5.3	
Not satisfied with the cleanliness of hospital ward/room	0.0	2.9
Not satisfied with the cleanliness of hospital toilets/bathrooms	2.3	7.0
Not satisfied with hospital food	13.9	

Doctors:		
Did not get understandable answers from doctors in response to important questions *#*	1.8	17.5
Did not have relationship of confidence or trust with the doctors	0.6	12.5
Why important tests were being done not explained in a way patient could understand	0.4	33.3
Important side effects of medications not explained in a way patient could understand *#*	13.5	49.1

Nurses:		
Did not get understandable answers from nurses in response to important questions *#*	3.5	33.3
Did not have relationship of confidence or trust with the nurses	2.9	25.7
There were not enough nurses on duty to care for the patient	3.5	14.7
On average, waited for help for more than 5 minutes after pressing call bell	12.3	

General Treatment and care:		
Patient received conflicting information from members of the medical team *#*	5.3	20.7
Not given enough information about condition and treatment	5.3	
Not involved in decisions about care and treatment as much as the patient wanted *#*	5.9	25.9
Family not given enough opportunity to talk to the doctor if they wanted to *#*	1.8	25.7
Did not find anyone on staff to talk to about worries and fears. *#*	20.0	27.6
Not given enough privacy during discussion about condition and treatment	2.3	12.9
Not given enough privacy during examination or treatment	1.2	8.8

Operations and Procedures:		
Risks/benefits of operations/procedures not explained in a way patient could understand	2.9	14.3
Not explained beforehand what would be done during the operation or procedure	2.9	29.0
Beforehand didn't get understandable answers to questions about the operation/procedure	5.7	32.1
Beforehand not told how to expect to feel after the operation/procedure	8.7	37.7
Process of anesthesia/pain control not explained in a way patient could understand	1.5	20.9
Not given understandable explanation about how the operation/procedure had gone	1.5	20.0

Overall care:		
Tests not carried out at their scheduled time	7.7	21.3
Not treated with respect and dignity during the hospital admission *#*	1.2	9.5
Doctors and nurses did not work very well together	0.6	
Never asked for views on the quality of care provided	68.6	
Thought he/she was not being charged fairly for their care and treatment	23.7	
Not knowing how much would eventually be paid worried the patient	13.0	30.2

# : Items included in the PPE-15 [13]

## Discussion

The concept of tailoring medical care towards patient expectations and the idea of patient-centeredness is new in developing countries. Our study is the first of its kind to be carried out in Pakistan since all previous studies have focused on specific areas of patient care such as the emergency department [9], day care surgery [10] or family medicine sections of the hospital [11]. On the other hand we have assessed patient's opinions about their general care and treatment, the degree of education imparted to them about their disease and treatment, the adequacy of communication with the medical team as well as issues of privacy and respect right from admission up till discharge. Furthermore, we have used the Urdu translation of a widely used internationally validated questionnaire [12] so as to allow us to compare our results with those of the west in a more reliable manner. However, it is worth pointing out that while most patient satisfaction studies, including those by the NHS, rely on response to mailed questionnaires, our questionnaire were filled by direct face to face interview. As pointed out by Labarere J et al [16], global satisfaction is higher in mail survey groups compared to face to face interview groups. In other words, mail survey often tends to overestimate patient satisfaction. This difference in the method of questionnaire filling must be kept in mind while drawing any meaningful comparisons between the results of our study and those of the NHS survey.

In the emergency section, patients were asked three questions dealing with information provided to them regarding their treatment and condition, provision of privacy and the time it took for them to get a bed in the ward. Of the patients who had been admitted from the ER, only 8% felt that they were not given enough information about their condition and their treatment. This is a very low number, and compares extremely favorably with a study focusing on the emergency department in Sweden [17] as well as the NHS survey carried out in 2004 [15], in which 20–23% of patients felt they were not given sufficient information regarding their diagnoses. However, we believe that this difference is primarily because of different expectations of Pakistani patients when compared to the west. Patients in Pakistan do not feel the need to question, or indeed learn about their condition and treatments as much as patients in the west where most patients would like to know even the finer details.

Among the other aspects of care assessed in the ER, only 6% of patients felt a complete lack of privacy while being managed, while nearly 41% were dissatisfied to some degree by the level of privacy afforded to them. When combined, this proportion is higher than in the west, where only 21–28 % of patients were dissatisfied with the level of privacy [18]. There could be two possible explanations to this. Firstly, the ethoses of extending privacy to the patients during examination and treatment have been primarily adopted from the west and doctors in this part of the world possess an attitude of paying much less emphasis on this area than their counterparts in the west. This is in spite of the fact that the society in Pakistan is more conservative and patients especially females prefer complete privacy. These two factors complement one another to produce a feeling of lack of privacy among patients. Furthermore, the level of privacy desired by Pakistani patients cannot always be assured in places like the emergency section. Finally, nearly half the patients (48%) felt they had to wait too long to get a bed in the hospital after presenting to the ER, a number which is comparable to other studies [15].

In the section on the wards, a number of patients reported being bothered by noise from other patients in the same ward (23%); however only 5% of patients claimed to be bothered by noise from the hospital staff. This may suggest that while the staff itself is careful not to disturb the patients, they do not play a proactive role in decreasing the noise created by other patients and their attendants. In our study very few patients reported any degree of dissatisfaction with the cleanliness of the wards or the washrooms. Our figures compare extremely favorably with data from the NHS survey [16] as well as from Brazil [19] where the respective proportions are much higher. Finally, 14% of patients were not happy with the quality of hospital food provided to them, which is better than the 46% of the NHS survey [15], but nevertheless a number that still needs improvement. The hospital provides standardized food to all patients along with special diets for patients with special needs. The food is generally kept low in spices and this along with the lack of selection on offer may be responsible for the dissatisfaction expressed by the patients.

Our study showed that patients generally had mixed views about the doctors treating them. Approximately 20 percent said they did not always receive an understandable answer in response to important questions put to the doctor while very few had this problem all the time. This figure is much higher compared to studies carried out in the United States [18] but comparable to a study carried out in Brazil [19], another developing country.

The second question in the ward section was regarding the patients having a relationship of confidence and trust with their attending doctors. In our study less than 1% of patient did not have the confidence or trust in their doctors while about twelve and a half percent trusted the doctors to a limited extent. When compared to other studies carried out in UK [15] our results are better but the lack of trust is relatively greater when compared to a similar study carried out in the US [18]. The system in UK is based on primary care while in our hospital as in the USA, it is regulated by the private sector. This could mean greater personal attention given by the doctor to the patient and thus explain this difference. The effect of cultural attitude and behavior towards the doctors can also not be disregarded when making such a comparison.

A significant majority reported that they were not told of the side effects of medications given to them or the rationale behind investigations performed. Although this figure is comparable to that reported in western literature [15], it still shows that there are gaps in patient education, and consent before every investigation is not necessarily obtained after thorough explanation of the reasons for doing so.

Nurses are an integral part of the health care system. Providing continuity of care is one of the many important jobs of a nurse. They are responsible for the daily physical comfort of the patient and routine tasks like taking the patient to the bathroom, bathing, providing proper meals, administering medications and attending to their calls. Furthermore nurses work as a team with other health care providers in carrying out orders such as getting tests done on time, administering and maintaining IV lines and answering important relevant questions.

Our study centered mainly on assessing patient satisfaction regarding nurses by asking their approval on nurse availability, ability to answer questions and overall trust and confidence of patients in the nursing team. The percentage of patients who never received clear answers to their questions from nurses was low as was the percentage who had complete lack of trust in the nursing team. These proportions are comparable or in instances even better than those reported in the west. However, if we also include the number of patients who only occasionally got clear answers or only had partial trust in the nurses, the two figures become considerably high and alarming. The inability to answers questions well enough can be attributed in part to a lack of knowledge of the nurses or the fact that patients may be asking questions from them that should normally be addressed to the doctor. It may also indicate the need for better communication between the doctors and nurses and for improvement in the communication skills of the nursing staff. The fact that nurses were not always trusted is mainly because patients in our part of the world do not regard nurses as knowledgeable healthcare providers and have yet to learn to take them into confidence regarding their disease and condition. However, most patients thought that doctors and nurses cooperated well during their treatment.

Our study shows that compared to developed nations [15,18], a much higher number of patients had to wait more than five minutes after pressing the call bell before a nurse attended to them. We regard that patients' call should be answered as soon as possible and any delay beyond five minutes should be held unacceptable. Many patients also thought that there were not enough nurses on duty all the time and this could partially be responsible for the delay in response to the call bell.

Most of the patients did not report receiving conflicting information from the members of the medical team. However 5.3% of the patients felt that they received conflicting information on many occasions while 20.7% reported this to be the case on very few occasions. Similarly, while most of the patients seemed satisfied by the knowledge imparted to them regarding their condition and treatment, 5.3% felt that they were not given enough information. This is a very low number, and compares extremely favorably with western figures [15], in which 23 % of patients felt that they were not given sufficient information about their condition and treatment. The percentage of patients who wanted greater involvement in their care was also less than in the west as were the proportions dissatisfied with the degree of privacy provided to them during examination and treatment [15,18,19]. These results mirror the responses given about information provided to them in the emergency and may be due to similar reasons.

Among other aspects, 1.8% of the patients felt that their families were not at all given enough opportunity to talk to the doctors, however 25.7% of the patients felt this to be the case to only some extent. When compared with statistics from UK [15] this result again matches favorably as 55% of the patients in their setting felt that their families were not given enough opportunities. Doctors in this part of the world have a greater tendency to involve the families owing to the cultural values of our society. However, this difference could very well be the result of different levels of expectations. Our figures in this sphere are comparable to those in USA [18].

Regarding another aspect, 20% of the patients did not find anyone in the staff to talk to about their worries and fears while 27.6% felt that they were given emotional support to only some extent. When compared with figures from NHS survey [15], our figures are much higher as only 10% of NHS patients felt that they did not find anyone in the hospital staff to talk to about their worries and fears. Since we feel there is no staff shortage in our hospital this difference may be because many doctors in our region only pay attention to the physical ailments of the patients and often neglect the adverse psychological factors associated with hospital admission.

One area where patients consistently reported dissatisfaction related to operations and procedures carried out during their stay. Very few reported that they had not at all been explained beforehand about what would happen during the procedure, or how to feel after the procedure was complete. However, as many as one third felt that the amount of information provided was not adequate. Many also complained that they did not get understandable answers to their questions about the procedure/operation and were not explained the risks/benefits of the procedure. This is despite the fact that all patients are required to sign a consent form before the procedure is performed. Our experience suggests that many patients sign this consent form without reading its content and the medical staff also does not make extra effort to encourage them to read the consent form. Furthermore, even when the consent form is orally read out to patients, many of the less educated patients might not be able to absorb the relevant information from what may be a complex consent form for them.

Up to one fifth of patients thought they were not provided enough information regarding anesthesia and pain control methods. A similar percentage complained that they were not told about the success/failure of the procedure at the end of the operation.

The first question about overall care was about whether the patients were treated with respect during their hospital stay. It was encouraging to know that most of the patients said that the staff and doctors treated them with respect. More than 98 % of the patients said that they were treated with respect and dignity. This was comparable to other international studies that reported that 88–92% [15,18,19] of their patients believed that they were treated with respect and dignity. 9.5% of the patients in our study said that they were generally satisfied with the amount of respect they got but they weren't fully satisfied.

In our study 29% of the patients said that tests were not being carried out at the appointed time. Studies in UK have reported similar figures [15]. The reason for this could be a lack of coordination between multiple departments of the institution or simply situations beyond the control of the care provider like giving preference to an emergency case.

An astounding 68.6% of the patients said that they were never asked for views on the quality of care provided. This is in spite of the fact that our institution keeps a suggestions box available at every reception unit and patients have the right to fill a complaint form as well. This figure shows that the unit staff as a whole was reluctant in putting direct questions to the patients regarding useful feedback since there is no in-built system in the hospital to ensure feedback from each and every patient.

A significant number of the patients thought that they were not being fairly charged for their treatment. Our institution is privately run and hence the cost of care is more expensive than government-run hospitals where medical care is heavily subsidized. We feel that most patients compare costs at our hospitals with those incurred in government hospitals coming forth with obvious conclusions.

Although our hospital does provide patients a rough estimate of the total expenses expected to be incurred on their stay, as many as 40.3% of the patients were worried at some point during the stay about how much they would have to pay eventually. In a study from USA [18] only 16.9% of the patients worried of not knowing how much to pay eventually. Our higher figure may be due to fact that in the west most patients have insurance coverage while in Pakistan most patients have to pay for their treatment on their own. This worry may also be in part because of the impression that they were being unfairly charged as explained above apart from some cases where patient stay might get extended and the expenses might shoot beyond the initial estimate provided.

### Limitations of the study

The study was carried out only in one tertiary care hospital of Karachi and therefore we may not be able to generalize its conclusions to the whole city. Furthermore, since the study was carried out on patients still admitted to the hospital, there might be a tendency to underreport unsatisfactory areas for fear of reprisal from the doctors. Lastly, all questions are subjective in nature and we have not used any objective tool to measure patient satisfaction. This limits our ability to compare our results with studies that use different questionnaires to assess patient satisfaction. Furthermore, we have not attempted to perform a trans-cultural validation of the translated questionnaire.

## Conclusion

Although there is significant room for improvement in all areas of care, several areas need to be paid particular attention. In particular, the waiting time in the emergency before getting a bed in the ward should be decreased by increasing the capacity of the hospital to deal with growing patient numbers. The quality of food needs to be improved, possibly by putting more options on offer. Patients should be provided more privacy during their treatment and nurses and doctors need to improve their communication with the patients. In particular, the health care team should provide more emotional support to the patient so that they have at least someone in the staff with whom they can share their fears and worries. Surgical teams should make sure that they explain all the risks and benefits to the patients and patiently listen and answer their questions before getting the consent form signed for every procedure. Finally, an effort should be made to ask patients about their views on the care provided in all instances rather than just giving them an option to do so.

## Competing interests

The author(s) declare that they have no competing interests.

## Authors' contributions

SZI participated in conceiving the idea of the study, made and pre-tested the Urdu version of the questionnaire and approved the final text. KSS participated in data collection and approved the final text. SAA collected data and participated in making and pretesting Urdu version of the questionnaire. SUA wrote the manuscript and approved the final text. KF carried out data analysis and participated in making and prestesting Urdu version of the questionnaire. MG helped conceive the idea of the study and carried out data collection. MOH collected data and approved the final text. SHH helped conceive the idea of the study and participated in writing the manuscript. MTS also helped conceive the idea of the study and participated in data analysis. HMK participated in data analysis and in writing the manuscript. OFJ collected data and participated in data analysis. All authors read and approved the final manuscript.

## Pre-publication history

The pre-publication history for this paper can be accessed here:



## Supplementary Material

Additional file 1 NHS Inpatient Survey Questionnaire 2005 – Picker Institute of Europe. An Urdu translation of this questionnaire was utilized for our study. It was developed by the Picker Institute of Europe for the NHS Inpatient Survey (UK) 2005. Picker Institute retains full copyright of this questionnaire and no part of it may be utilized without their prior permission. Click here for file
