# Supplementary material for: Patients' satisfaction and opinions of their experiences during admission in a tertiary care hospital in Pakistan – a cross sectional study
Source: BMC Health Serv Res. 2007 Oct 3;7:161. doi: 10.1186/1472-6963-7-161 (PMC2082029; doi:10.1186/1472-6963-7-161)
Supplement: Additional file 1 — NHS Inpatient Survey Questionnaire 2005 – Picker Institute of Europe. An Urdu translation of this questionnaire was utilized for our study. It was developed by the Picker Institute of Europe for the NHS Inpatient Survey (UK) 2005. Picker Institute retains full copyright of this questionnaire and no part of it may be utilized without their prior permission. [file 1472-6963-7-161-S1.pdf]

INPATIENT QUESTIONNAIRE

What is the survey about?

This survey is about your **most recent** experience as an **inpatient** at the National Health Service hospital named in the letter enclosed with this questionnaire.

Who should complete the questionnaire?

The questions should be answered by the person named on the front of the envelope. If that person needs help to complete the questionnaire, the answers should be given from his/her point of view – not the point of view of the person who is helping.

Completing the questionnaire

For each question please tick clearly inside one box using a black or blue pen.

Sometimes you will find the box you have ticked has an instruction to go to another question. By following the instructions carefully you will miss out questions that do not apply to you.

Don't worry if you make a mistake; simply cross out the mistake and put a tick in the correct box.

Please **do not** write your name or address anywhere on the questionnaire.

Questions or help?

If you have any queries about the questionnaire, please call the helpline number given in the letter enclosed with this questionnaire.

Taking part in this survey is voluntary. **Your answers will be treated in confidence.**

Picker Institute Europe. Copyright 2005. All rights reserved. Inpatient core questionnaire v10 09/08/05

Please remember, this questionnaire is about your **most recent** stay at the hospital named in the accompanying letter.

ADMISSION TO HOSPITAL

1. Was your most recent hospital stay planned in advance or an emergency?

1 ☐ Emergency or urgent ➔ **Go to Question 2**

2 ☐ Waiting list or planned in advance ➔ **Go to Question 12**

3 ☐ Something else ➔ **Go to Question 2**

EMERGENCY CARE

2. Did you travel to the hospital by ambulance?

1 ☐ Yes ➔ **Go to Question 3**

2 ☐ No ➔ **Go to Question 7**

3. Were the ambulance crew reassuring?

1 ☐ Yes, definitely

2 ☐ Yes, to some extent

3 ☐ No

4 ☐ Don't know / Can't remember

4. Did the ambulance crew explain your care and treatment in a way you could understand?

1 ☐ Yes, definitely

2 ☐ Yes, to some extent

3 ☐ No

4 ☐ Don't know / Can't remember

5. Did the ambulance crew do everything they could to help control your pain?

1 ☐ Yes, definitely

2 ☐ Yes, to some extent

3 ☐ No

4 ☐ I did not have any pain

6. Overall, did the ambulance crew treat you with respect and dignity?

1 ☐ Yes, definitely

2 ☐ Yes, to some extent

3 ☐ No

4 ☐ Don't know / Can't remember

THE EMERGENCY DEPARTMENT

7. When you arrived at the hospital, did you go to the Emergency Department (Casualty /A&E / Medical or Surgical Admissions unit)?

1 ☐ Yes ➔ **Go to Question 8**

2 ☐ No ➔ **Go to Question 12**

8. Did you think the order in which patients were seen in the Emergency Department was fair?

1 ☐ Yes

2 ☐ No

3 ☐ Can't say / Don't know

Picker Institute Europe. Copyright 2005. All rights reserved.

9. While you were in the Emergency Department, how much information about your condition or treatment was given to you?

1 ☐ Not enough

2 ☐ Right amount

3 ☐ Too much

4 ☐ I was not given any information about my treatment or condition

10. Were you given enough privacy when being examined or treated in the Emergency Department?

1 ☐ Yes, definitely

2 ☐ Yes, to some extent

3 ☐ No

11. Following arrival at the hospital, how long did you wait before being admitted to a bed on a ward?

1 ☐ Less than 1 hour

2 ☐ At least 1 hour but less than 2 hours

3 ☐ At least 2 hours but less than 4 hours

4 ☐ At least 4 hours but less than 8 hours

5 ☐ 8 hours or longer

6 ☐ Can't remember

7 ☐ I did not have to wait

EMERGENCY PATIENTS, now please go to Question 17

WAITING LIST & PLANNED ADMISSION PATIENTS, please continue to Question 12

WAITING LIST OR PLANNED ADMISSION

12. Were you given a choice of **admission dates**?

1 ☐ Yes

2 ☐ No

3 ☐ Don't know / Can't remember

13. Overall, from the time you were first told you needed to be admitted to hospital, how long did you wait to be admitted?

1 ☐ Up to 1 month

2 ☐ 1 to 3 months

3 ☐ 3 to 6 months

4 ☐ 6 to 9 months

5 ☐ More than 9 months

6 ☐ Don't know / Can't remember

14. How do you feel about the length of time you were on the waiting list before your admission to hospital?

1 ☐ I was admitted as soon as I thought was necessary

2 ☐ I should have been admitted a bit sooner

3 ☐ I should have been admitted a lot sooner

Picker Institute Europe. Copyright 2005. All rights reserved. Inpatient core questionnaire v10 09/08/05

15. When you were told you would be going into hospital, were you given enough notice of your date of admission?

1 ☐ Yes, enough notice

2 ☐ No, not enough notice

16. Was your admission date changed by the hospital?

1 ☐ No

2 ☐ Yes, once

3 ☐ Yes, 2 or 3 times

4 ☐ Yes, 4 times or more

ALL TYPES OF ADMISSION

17. From the time you arrived at the hospital, did you feel that you had to wait a long time to get to a bed on a ward?

1 ☐ Yes, definitely

2 ☐ Yes, to some extent

3 ☐ No

THE HOSPITAL AND WARD

18. During your stay in hospital, did you ever share a room or bay with patients of the opposite sex?

1 ☐ Yes

2 ☐ No

19. Were you ever bothered by noise at night from **other patients**?

1 ☐ Yes

2 ☐ No

20. Were you ever bothered by noise at night from **hospital staff**?

1 ☐ Yes

2 ☐ No

21. In your opinion, how clean was the hospital room or ward that you were in?

1 ☐ Very clean

2 ☐ Fairly clean

3 ☐ Not very clean

4 ☐ Not at all clean

22. How clean were the toilets and bathrooms that you used in hospital?

1 ☐ Very clean

2 ☐ Fairly clean

3 ☐ Not very clean

4 ☐ Not at all clean

5 ☐ I did not use a toilet or bathroom

23. How would you rate the hospital food?

1 ☐ Very good

2 ☐ Good

3 ☐ Fair

4 ☐ Poor

5 ☐ I did not have any hospital food

Picker Institute Europe. Copyright 2005. All rights reserved. Inpatient core questionnaire v10 09/08/05

DOCTORS

24. When you had important questions to ask a doctor, did you get answers that you could understand?

1 ☐ Yes, always
2 ☐ Yes, sometimes
3 ☐ No
4 ☐ I had no need to ask

25. Did you have confidence and trust in the doctors treating you?

1 ☐ Yes, always
2 ☐ Yes, sometimes
3 ☐ No

26. Did doctors talk in front of you as if you weren't there?

1 ☐ Yes, often
2 ☐ Yes, sometimes
3 ☐ No

27. As far as you know, did doctors wash or clean their hands between touching patients?

1 ☐ Yes, always
2 ☐ Yes, sometimes
3 ☐ No
4 ☐ Don't know / Can't remember

NURSES

28. When you had important questions to ask a nurse, did you get answers that you could understand?

1 ☐ Yes, always
2 ☐ Yes, sometimes
3 ☐ No
4 ☐ I had no need to ask

29. Did you have confidence and trust in the nurses treating you?

1 ☐ Yes, always
2 ☐ Yes, sometimes
3 ☐ No

30. Did nurses talk in front of you as if you weren't there?

1 ☐ Yes, often
2 ☐ Yes, sometimes
3 ☐ No

31. In your opinion, were there enough nurses on duty to care for you in hospital?

1 ☐ There were always or nearly always enough nurses
2 ☐ There were sometimes enough nurses
3 ☐ There were rarely or never enough nurses

Picker Institute Europe. Copyright 2005. All rights reserved. Inpatient core questionnaire v10 09/08/05

32. As far as you know, did nurses wash or clean their hands between touching patients?

1 ☐ Yes, always
2 ☐ Yes, sometimes
3 ☐ No
4 ☐ Don't know / Can't remember

YOUR CARE AND TREATMENT

33. Sometimes in a hospital, a member of staff will say one thing and another will say something quite different. Did this happen to you?

1 ☐ Yes, often
2 ☐ Yes, sometimes
3 ☐ No

34. Were you involved as much as you wanted to be in decisions about your care and treatment?

1 ☐ Yes, definitely
2 ☐ Yes, to some extent
3 ☐ No

35. How much information about your condition or treatment was given to **you**?

1 ☐ Not enough
2 ☐ The right amount
3 ☐ Too much

36. If your family or someone else close to you wanted to talk to a doctor, did they have enough opportunity to do so?

1 ☐ Yes, definitely
2 ☐ Yes, to some extent
3 ☐ No
4 ☐ No family or friends were involved
5 ☐ My family did not want or need information
6 ☐ I did not want my family or friends to talk to a doctor

37. Did you find someone on the hospital staff to talk to about your worries and fears?

1 ☐ Yes, definitely
2 ☐ Yes, to some extent
3 ☐ No
4 ☐ I had no worries or fears

38. Were you given enough privacy when discussing your condition or treatment?

1 ☐ Yes, always
2 ☐ Yes, sometimes
3 ☐ No

39. Were you given enough privacy when being examined or treated?

1 ☐ Yes, always
2 ☐ Yes, sometimes
3 ☐ No

40. Did you get enough help from staff to eat your meals?

1 ☐ Yes, always

2 ☐ Yes, sometimes

3 ☐ No

4 ☐ I did not need help to eat meals

41. How many minutes after you used the call button did it usually take before you got the help you needed?

1 ☐ 0 minutes/right away

2 ☐ 1-2 minutes

3 ☐ 3-5 minutes

4 ☐ More than 5 minutes

5 ☐ I never got help when I used the call button

6 ☐ I never used the call button

PAIN

42. Were you ever in any pain?

1 ☐ Yes ➔ **Go to Question 43**

2 ☐ No ➔ **Go to Question 44**

43. Do you think the hospital staff did everything they could to help control your pain?

1 ☐ Yes, definitely

2 ☐ Yes, to some extent

3 ☐ No

OPERATIONS & PROCEDURES

44. During your stay in hospital, did you have an operation or procedure?

1 ☐ Yes ➔ **Go to Question 45**

2 ☐ No ➔ **Go to Question 52**

45. Beforehand, did a member of staff explain the risks and benefits of the operation or procedure in a way you could understand?

1 ☐ Yes, completely

2 ☐ Yes, to some extent

3 ☐ No

4 ☐ I did not want an explanation

46. Beforehand, did a member of staff explain what would be done during the operation or procedure?

1 ☐ Yes, completely

2 ☐ Yes, to some extent

3 ☐ No

4 ☐ I did not want an explanation

47. Beforehand, did a member of staff answer your questions about the operation or procedure in a way you could understand?

1 ☐ Yes, completely

2 ☐ Yes, to some extent

3 ☐ No

4 ☐ I did not have any questions

Picker Institute Europe. Copyright 2005. All rights reserved. Inpatient core questionnaire v10 09/08/05

48. Beforehand, were you told how you could expect to feel after you had the operation or procedure?

1 ☐ Yes, completely

2 ☐ Yes, to some extent

3 ☐ No

49. Before the operation or procedure, were you given an anaesthetic to put you to sleep or control your pain?

1 ☐ Yes ➔ **Go to Question 50**

2 ☐ No ➔ **Go to Question 51**

50. Before the operation or procedure, did the anaesthetist explain how he or she would put you to sleep or control your pain in a way you could understand?

1 ☐ Yes, completely

2 ☐ Yes, to some extent

3 ☐ No

51. After the operation or procedure, did a member of staff explain how the operation or procedure had gone in a way you could understand?

1 ☐ Yes, completely

2 ☐ Yes, to some extent

3 ☐ No

LEAVING HOSPITAL

52. On the day you left hospital, was your discharge delayed for any reason?

1 ☐ Yes ➔ **Go to Question 53**

2 ☐ No ➔ **Go to Question 55**

53. What was the **MAIN** reason for the delay? (Tick ONE only)

1 ☐ I had to wait for **medicines**

2 ☐ I had to wait to **see the doctor**

3 ☐ I had to wait for an **ambulance**

4 ☐ Something else

54. How long was the delay?

1 ☐ Up to 1 hour

2 ☐ Longer than 1 hour but no longer than 2 hours

3 ☐ Longer than 2 hours but no longer than 4 hours

4 ☐ Longer than 4 hours

55. Did a member of staff explain the purpose of the medicines you were to take at home in a way you could understand?

1 ☐ Yes, completely ➔ **Go to Question 56**

2 ☐ Yes, to some extent ➔ **Go to Question 56**

3 ☐ No ➔ **Go to Question 56**

4 ☐ I did not need an explanation ➔ **Go to Question 56**

5 ☐ I had no medicines ➔ **Go to Question 58**

Picker Institute Europe. Copyright 2005. All rights reserved. Inpatient core questionnaire v10 09/08/05

56. Did a member of staff tell you about medication side effects to watch for when you went home?

1 ☐ Yes, completely
2 ☐ Yes, to some extent
3 ☐ No
4 ☐ I did not need an explanation

57. Were you given clear written information about your medicines?

1 ☐ Yes, completely
2 ☐ Yes, to some extent
3 ☐ No
4 ☐ Don't know / Can't remember

58. Did a member of staff tell you about any danger signals you should watch for after you went home?

1 ☐ Yes, completely
2 ☐ Yes, to some extent
3 ☐ No
4 ☐ It was not necessary

59. Did the doctors or nurses give your family or someone close to you all the information they needed to help you recover?

1 ☐ Yes, definitely
2 ☐ Yes, to some extent
3 ☐ No
4 ☐ No family or friends were involved
5 ☐ My family or friends did not want or need information

60. Did hospital staff tell you who to contact if you were worried about your condition or treatment after you left hospital?

1 ☐ Yes
2 ☐ No
3 ☐ Don't know / Can't remember

61. Did you receive copies of letters sent between hospital doctors and your family doctor (GP)?

1 ☐ Yes, I received copies
2 ☐ No, I did not receive copies
3 ☐ Not sure/ don't know

OVERALL

62. Overall, did you feel you were treated with respect and dignity while you were in the hospital?

1 ☐ Yes, always
2 ☐ Yes, sometimes
3 ☐ No

63. How would you rate how well the doctors and nurses worked together?

1 ☐ Excellent
2 ☐ Very good
3 ☐ Good
4 ☐ Fair
5 ☐ Poor

Picker Institute Europe. Copyright 2005. All rights reserved. Inpatient core questionnaire v10 09/08/05

64. Overall, how would you rate the care you received?

1 ☐ Excellent

2 ☐ Very good

3 ☐ Good

4 ☐ Fair

5 ☐ Poor

65. During your hospital stay, were you ever asked to give your views on the quality of your care?

1 ☐ Yes

2 ☐ No

3 ☐ Don't know / Can't remember

ABOUT YOU

66. Are you male or female?

1 ☐ Male

2 ☐ Female

67. What was your year of birth?

(Please write in) e.g. | 1 | 9 | 3 | 4 |

68. How old were you when you left full-time education?

1 ☐ 16 years or less

2 ☐ 17 or 18 years

3 ☐ 19 years or over

4 ☐ Still in full-time education

69. Overall, how would you rate your health during the **past 4 weeks**?

1 ☐ Excellent

2 ☐ Very good

3 ☐ Good

4 ☐ Fair

5 ☐ Poor

6 ☐ Very poor

70. Do you have a long-standing physical or mental health problem or disability?

1 ☐ Yes ➔ **Go to 71**

2 ☐ No ➔ **Go to 72**

71. Does this problem or disability affect your day-to-day activities?

1 ☐ Yes, definitely

2 ☐ Yes, to some extent

3 ☐ No

Picker Institute Europe. Copyright 2005. All rights reserved. Inpatient core questionnaire v10 09/08/05

72. To which of these ethnic groups would you say you belong? (**Tick ONE only**)

a. WHITE

1 ☐ British

2 ☐ Irish

3 ☐ Any other White background **(Please write in box)**

b. MIXED

4 ☐ White and Black Caribbean

5 ☐ White and Black African

6 ☐ White and Asian

7 ☐ Any other Mixed background **(Please write in box)**

c. ASIAN OR ASIAN BRITISH

8 ☐ Indian

9 ☐ Pakistani

10 ☐ Bangladeshi

11 ☐ Any other Asian background **(Please write in box)**

d. BLACK OR BLACK BRITISH

12 ☐ Caribbean

13 ☐ African

14 ☐ Any other Black background **(Please write in box)**

e. CHINESE OR OTHER ETHNIC GROUP

15 ☐ Chinese

16 ☐ Any other ethnic group **(Please write in box)**

Picker Institute Europe. Copyright 2005. All rights reserved.

OTHER COMMENTS

If there is anything else you would like to tell us about your experiences in the hospital, please do so here.

Was there anything particularly good about your hospital care?

Was there anything that could be improved?

Any other comments?

THANK YOU VERY MUCH FOR YOUR HELP

Please check that you answered all the questions that apply to you.

Please post this questionnaire back in the FREEPOST envelope provided.

No stamp is needed.
